# Motor interactions with another person: do individuals with Autism Spectrum Disorder plan ahead?

**DOI:** 10.3389/fnint.2013.00023

**Published:** 2013-04-17

**Authors:** David A. Gonzalez, Cheryl M. Glazebrook, Breanna E. Studenka, Jim Lyons

**Affiliations:** ^1^Department of Kinesiology, University of WaterlooWaterloo, ON, Canada; ^2^Department of Cognitive Neurology, Sunnybrook Health Sciences CentreToronto, ON, Canada; ^3^Faculty of Kinesiology and Recreation Management, Health, Leisure, and Human Performance Research Institute, University of ManitobaWinnipeg, MB, Canada; ^4^Department of Health, Physical Education and Recreation, Utah State UniversityLogan, UT, USA; ^5^Department of Kinesiology, McMaster UniversityHamilton, ON, Canada

**Keywords:** Autism Spectrum Disorder, motor skills, movement planning, theory of mind (ToM), joint-action

## Abstract

Interpersonal motor interactions (joint-actions) occur on a daily basis. In joint-action situations, typically developing (TD) individuals consider the end-goal of their partner and adjust their own movements to accommodate the other person. The movement planning processes required for joint-action may, however, be difficult for individuals with an Autism Spectrum Disorder (ASD) given documented difficulties in performance on theory of mind (ToM) and motor tasks. The goal of this experiment was to determine if individuals with ASD exhibit end-state comfort behaviors similar to their TD peers in joint-action situations. Participants were asked to either pass, place, or use three common tools: a wooden toy hammer, a stick, or a calculator. These tools were selected because the degree of affordance they offer (i.e., the physical characteristics they posses to prompt proper use) ranges from direct (hammer) to indirect (calculator). Participants were asked to pass the tool to a confederate who intended to place the tool down, or use the tool. Variables of interest included beginning and end-state grip orientations of the participant and confederate (comfortable or uncomfortable) as a function of task goal, and the side to which the tool was placed or passed. Similar to Gonzalez et al. (2011), some individuals with ASD maximized their partner's beginning-state comfort by adopting personally uncomfortable postures. That said, their performance was more variable than their TD peers who consistently passed tools in a manner that facilitated comfortable use by the confederate. Therefore, the movement planning processes used to prepare to pass a tool are not stereotypical across all individuals with ASD. We propose that the novel joint-action task described herein provides the basis for testing an important link between motor performance and more complex social and communication behaviors.

## Introduction

Not only is the coordination between our own joints and limbs very complex, many daily tasks require us to coordinate our actions with another individual, further increasing task complexity. Success in a number of sports also depends on the precision of coordination between two or more individuals (e.g., rowing, synchronized diving). Although most of us will not attempt such feats, we do have to coordinate movements with others to achieve many goals in our everyday lives. This type of coordination (often referred to as joint-action) requires us to understand the perspective of another person; or at the very least, to have a sense of the common goal, as well as a shared understanding of how to achieve this goal. These everyday interactions appear simple or straightforward, however, the complexity of interpersonal coordination becomes apparent for individuals who exhibit difficulties with social interaction. By definition, individuals with an autism spectrum disorder (ASD) have difficulty with social and communication behaviors (American Psychiatric Association, 2000). Beyond the delays in social and communication skills, there are also documented differences in how individuals with ASD perform motor, imitation, and executive function tasks (Fournier et al., 2010; Kana et al., 2011; Vanvuchelen et al., 2011; Brown and Bebko, 2012). However, little is known about how individuals with ASD perform motor skills when the motor task requires interaction with another person. A joint-action task provides a unique opportunity to assess both movement planning and non-verbal communication behaviors exhibited by individuals with ASD.

In order to interact gracefully with an object or another person, one needs to be able to incorporate characteristics of those objects and persons into their action plans. One elegant approach to assess movement planning was first introduced by Rosenbaum and Jorgensen (1992). They suggested that movements are planned such that maximal comfort and stability are achieved with the terminal posture (the End-State Comfort Effect). Of greater interest was the observation that, in order to achieve “end-state comfort,” participants will almost always forego a comfortable starting posture in order to achieve a comfortable end posture. This type of behavior is indicative of efficient forward planning, as the person must think ahead to the terminal requirements of the movement to understand that the initial discomfort will ultimately lead to having a comfortable posture when using the object. Other researchers have consistently reported an end-state comfort effect in a variety of scenarios (Haggard, 1998; Cohen and Rosenbaum, 2004; Weigelt et al., 2006).

The ability to plan for end-state comfort is less clear for individuals with ASD. van Swieten et al. (2010) asked participants to grasp a dowel and were asked to match the position of a dowel on a computer screen using either a clockwise or counter clockwise movement. van Swieten et al. (2010), reported that children with ASD chose postures that led to end-state comfort about 50% of the time, which was not different than the age-matched controls (9–14 years-old). This would suggest that individuals with ASD are able to plan some motor actions to ensure a comfortable end-state posture. However, Hughes (1996) demonstrated that 12–13 year-old children with ASD transported a painted dowel using an underhand grip as opposed to the overhand grip used by younger (3–4 year-old), typically developing (TD) children. The underhand grip resulted in beginning-state comfort, but in many cases led to an uncomfortable end-state posture, indicating a lack of action planning. Conflicting results in these types of tasks are not uncommon. Indeed a number of studies have reported atypical movement planning processes in participants with ASD across a variety of contexts. One consistent finding across younger and older children with ASD, as well as young adults, is more variable reaction times for simple goal-directed reaching movements (Glazebrook et al., 2006, 2009; Rinehart et al., 2006; Dowd et al., 2012). These authors have suggested that the greater variability, and in some cases longer duration, of reaction time reflects aberrant movement planning processes. For example, individuals with ASD exhibit greater within-person spatial and temporal variability early in the execution of goal-directed reaching movements. The observed differences in early online control are consistent with atypical movement planning processes (Glazebrook et al., 2009; Elliott et al., 2010). Although slower and more variable, young adults with ASD are successful using direct visual cues about hand and direction. As the task requirements are increased, however, the difficulty with movement planning becomes more apparent (Glazebrook et al., 2008; Nazarali et al., 2009; Dowd et al., 2012).

Greater variability (both within and between individuals) in the movements produced by individuals with ASD could be due to the abnormal connections between brain regions that ultimately lead to impairments in internal models of action, as well as in understanding the associated intentions of others (Mostofsky and Ewen, 2011). Mostofsky and Ewen (2011) suggest, as have others (Beilin and Fireman, 1999), that internal models of intended actions are important in movement planning as well as in understanding the intentions of others' actions. In other words, to understand the actions of another, one needs to know what the consequences of those actions will be. However, if there are inconsistencies in internal models of actions, assessed consequences of those actions may also be inconsistent, leading to difficulties interacting with other individuals. Indeed, there is a growing body of literature supporting the idea that coordination of movements across participants does occur (Welsh et al., 2005, 2007). Within that literature there are also a few examples of how individuals work together to attain a common goal (see Marsh et al., 2009, for a review).

ToM tasks are widely used in the ASD literature to test whether individuals can understand the perspective of another (Ozonoff et al., 1991; Pellicano, 2007). In the classic paradigm, Baron-Cohen et al. (1985), reported that individuals with ASD do not comprehend why Sally would look for a marble where she had left it; instead they believe Sally would look for the marble in the location that Anne moved it to (but Sally had not seen). Although individuals with ASD can learn to solve basic ToM tasks such as this, Ozonoff et al. (1991) reported that when ToM tasks become more complex individuals with ASD begin to demonstrate deficits. For example, the performance of the individuals with ASD was similar to their TD peers when they were asked to put a series of pictures into a sequence that tells a story, but only in situations where the story did not require mental state attributions, (e.g., *knowing* that an object is light when it superficially *looks* heavy) (Ozonoff et al., 1991; Pellicano, 2007). A similar pattern of performance is observed when comparing literal and figurative language (MacKay and Shaw, 2004; Pellicano, 2007). Likewise, Boria et al. (2009) reported that participants with ASD had no difficulty inferring why someone was grasping an object when the grasp was accompanied by functional information about the action (e.g., paper scraps to indicate the action of cutting), but had marked difficulty inferring why someone was grasping an object based on the characteristics of the posture alone (e.g., when a phone was grasped on the side to move it or on the receiver to answer it). In summary, when they are successful, individuals with ASD appear to use different strategies to solve the ToM tasks, and although this allows some success, the altered strategies do not lead to natural performance and prevent application to more complex scenarios.

**Figure 1:**
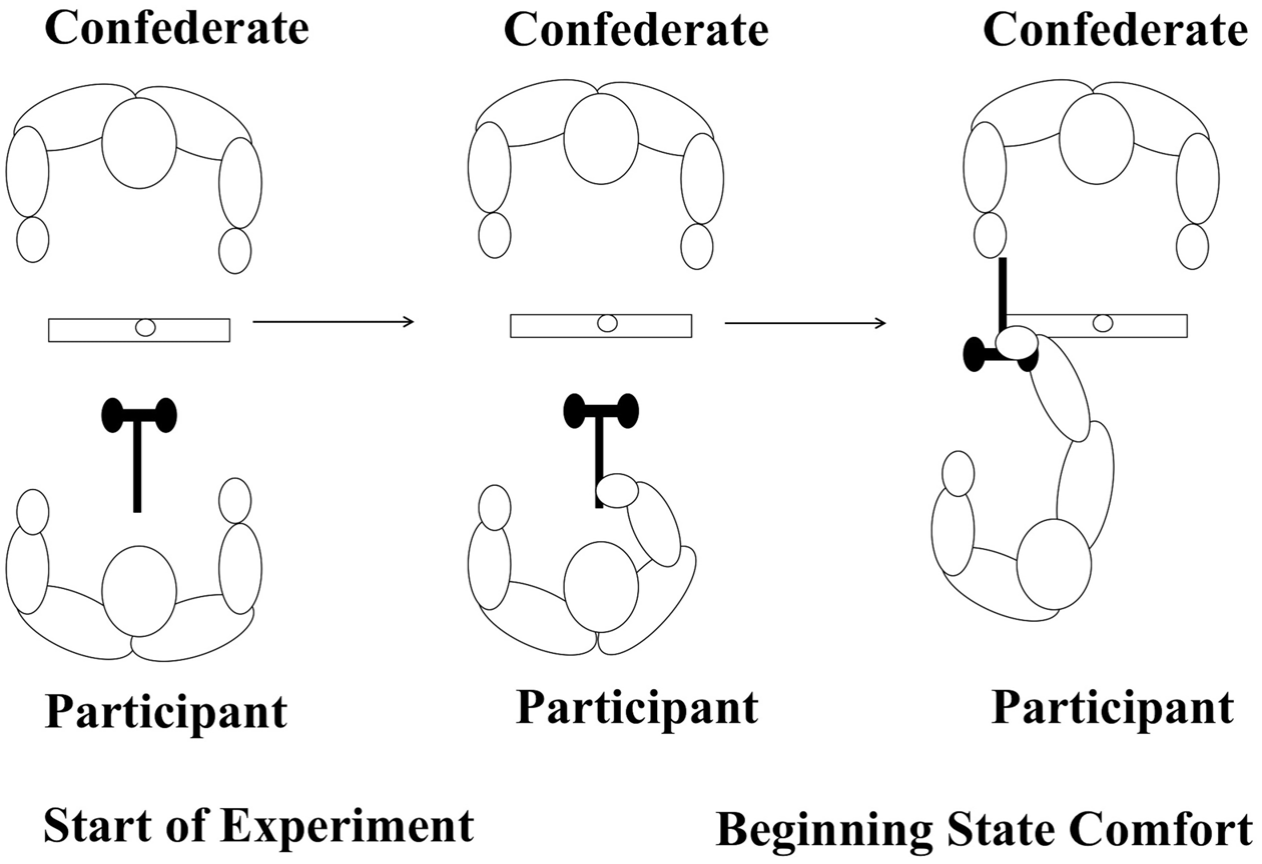
**Participants grasping and turning the hammer (placed in a comfortable position relative to the participant) to give the confederate beginning-state comfort**.

One potential limitation of most ToM studies is that typically the tasks used are inherently verbal in nature, and/or do not involve real-time interaction with another person. As such, it is unclear whether results from these studies are truly indicative of difficulties in considering the perspective of others, or if they reflect more generalized difficulties putting that perspective into words. Recently, we (Gonzalez et al., 2011) developed a motor ToM paradigm to assess how individuals prepare non-verbal actions when they are asked to consider the movement goals of another person. That is, we adopted a joint-action protocol wherein participants were assessed on whether they anticipated which action plan results in a beneficial beginning-state posture for their partner's movement (see Figure 1). Overall the results were remarkably consistent in that participants almost invariably considered the perspective of the second person by first anticipating that person's ultimate action goal and then facilitating the execution of that goal by passing the tool in a manner that maximized both the comfort and efficiency of the confederate's movement (e.g., handle first). Ray and Welsh (2011) also reported similar findings with TD participants. Consistent with Gonzalez et al. (2011), Ray and Welsh (2011) reported that participants passed the jug in a manner that facilitated the beginning-state comfort of the other person (handle facing the person) 86% of the time, even though it meant the participant could not hold the handle him/herself to pass the jug. Joint-action tasks that involve real-time interaction may be a new window into understanding how people with ASD understand and interpret the perspectives of another person.

Given the documented differences in movement planning and joint-action tasks, we were interested in how individuals with ASD perform a joint-action task when they have the opportunity to consider the perspective of another person. In other words, we tested a novel ToM task that requires a motor, as opposed to a verbal, response. Furthermore, according to research that indicates individuals with ASD are better able to understand movements related to a specific grasp posture when that posture is presented within a functional context (Boria et al., 2009), we hypothesized that individuals with ASD might be better able to infer the proper way to hand an object to another individual if the object primed the action to be performed (e.g., hammer for hammering vs. stick for hammering). Therefore, we aimed to determine if interpersonal deficits seen in non-motoric interactions of persons with ASD carry over to the task of inferring the intentions of another person when those intentions are related to a specific motor action. In order to accomplish this, we replicated Gonzalez et al.'s (2011) joint-action paradigm with a similar group of individuals with ASD. We predicted that the participants with ASD would perform their actions with more consideration for the actions of the confederate when the tool better primed the action to be performed by the confederate. More specifically, when the task was hammering, we expected participants with ASD to adjust their posture more readily to facilitate the beginning-state grasp of the confederate when handing the hammer vs. the stick because the action associated with the hammer was more concrete.

## Materials and methods

### Participants

Ten participants with an ASD (1 female; 2 left-handed males) participated in the present study. The mean chronological age of the participants with ASD was 32.7 years (*SD* = 10.8). Note that the participant demographics are consistent with Gonzalez et al. (2011), where the mean age of the 10 participants was 32.2 years (*SD* = 11.1); 1 female and 2 left-handed males. All 10 participants in the present study were diagnosed by a qualified health professional (3 were diagnosed with Asperger's syndrome). Participants completed the Peabody Picture Vocabulary Test-Revised and Raven's Progressive Matrices as a measure of verbal and non-verbal abilities respectively. Verbal age scores ranged from 3 to 27 years with a mean of 14 years (*SD* = 8.3). IQ equivalent scores of performance on Raven's Progressive Matrices ranged from 60 to 110 with a mean 84 (*SD* = 17). Table 1 illustrates individual participant demographics. In addition, participants reported taking one or more of the following medications: *Anafranil, Rispirdal, Adovan, Divalproex, Fluoxetine, Adderall, Carbamazepine, Citalopram, and Sertraline*. Participants were remunerated $5 for their participation. The experiment and procedure were approved by the McMaster University Human Ethics Board.

**Table 1:** **Participant demographics**.

**Participant**	**Sex**	**Age**	**Handedness**	**Verbal age**	**IQ equivalent**
1	Male	44	Right	12	94
2	Female	22	Right	9	74
3	Male	22	Right	15	79
4	Male	25	Left	15	90
5	Male	55	Right	27	110
6	Male	26	Right	3	78
7	Male	32	Left	14	60
8	Male	30	Right	3	82
9	Male	30	Right	27	76
10	Male	41	Right	16	100

### Apparatus

Individuals were provided with a calculator, a toy hammer, and a stick painted half white and half black. The different colors allowed for instructions in using the stick (which side to use as the handle and which to use as the hammer). The handle of the hammer was 2.1 cm in diameter and 14.8 cm in length, and the hexagonal head was 3.2 cm in length, 5.9 cm in width, and 3 cm in depth. The calculator was 8 cm wide × 15.5 cm long × 1.5 cm thick. The stick was 2.2 cm in diameter and 18.2 cm in length. A peg board with one peg sticking up (2.3 cm in diameter, 6 cm in length) was placed in front of a participant ~20 cm away from the front edge of the table (~67 cm high). Two 21.59 × 27.94 cm sheets of paper were placed on the right and left of the peg board. The tools and setup were the same as those used in the previous publication (Gonzalez et al., 2011).

The interactions with the tools were videotaped using a Panasonic MiniDV camera which allowed the researchers to score the data *post-hoc*.

### Procedure

Tasks not involving the confederate (self-tasks) were always performed before the tasks that involved a confederate (other tasks) in order to allow the participants to gain some experience with the tasks before having to interact with another person. The entire procedure took ~30 min to complete.

#### Self-task

Participants were seated throughout the entire procedure. All of the tools (hammer, calculator, and stick) were presented before the start of the experiment to allow familiarity. In the experimental session participants were presented with twelve different conditions: 3 Tool (hammer, calculator, stick) × 2 Orientation (comfortable, uncomfortable) × 2 Action (use, place) in a pseudorandom order. The pseudorandom order consisted of all the trials of each condition (e.g., tool: hammer; initial orientation: comfortable; action: use) being presented in a blocked fashion to provide participants an opportunity to develop strategies; however the order of the 12 conditions was counterbalanced across participants.

The participants were asked to either place or use the tool placed in front of them. That is, participants were asked to *use* the hammer, or the stick to *hammer* the peg, or to *use* the calculator to *calculate* a simple mathematical procedure (e.g., 62 × 17). The instructions were identical to that of Gonzalez et al. (2011). On some trials the participants were asked to place the tool on one of the sheets of paper, but which of the two sheets (the left or right) the participant placed the tool on was not specified. The tools were initially placed either in a comfortable (handle facing participant) or an uncomfortable (handle facing away from participant) orientation. We manipulated the initial orientation of the tool in order to assess if participants planned their own actions in manner that facilitated a comfortable end posture (i.e., end-state comfort).

The instructions for the action were given after the tool was placed in front of the participant (e.g., use the calculator to calculate 14 × 26). For the stick, which color they should use to hammer with was specified (e.g., hammer the peg with the black end). Each condition was presented six times, for a total of 72 trials for the self-tasks.

#### Other task

Each participant was asked to help the other individual (confederate) complete the same tasks. At the beginning of the experiment the experimenter mentioned that the confederate was right-handed and that the participants should make the task *as easy and efficient for the confederate as possible*. The confederate was an age appropriate male (28 years-old) and was consistent for all participants. Twenty-four different conditions were included: 3 Tool (hammer, calculator, stick) × 2 Participant Action (place tool, hand tool) × 2 Orientation (comfortable, uncomfortable) × 2 Confederate Action (use, place). The participants performed 6 trials per condition for a total of 144 trials for the other tasks. Participants were always given prior knowledge of which condition was to be performed for the upcoming trial. The same pseudorandom procedure employed in the self-task was used in the working with other task (i.e., blocking all trials of each condition, and randomly presenting the conditions).

On each trial the participant was told to give the tool to the confederate so that he could either use or place the tool. The participant was asked to either hand the tool directly to the confederate or to place the tool on one of the sheets provided so that the confederate could pick it up. The crucial condition occurred when the object had to be manipulated by the participant in order for the confederate to achieve beginning-state comfort (comfortable tool orientation). We included this condition because we were interested in determining if participants understood that the confederate would have an easier time using the tool if he was given the tool in a fashion that maximized his *beginning-state comfort* (i.e., grabbing the tool with a comfortable posture that required no manipulation to use the tool). The condition for each trial was predetermined by the experimenter who gave the instructions to both the confederate and the participant. The different conditions allowed for comparison of how the participants behaved when handing a tool to the confederate when the tool would be used vs. when the tool was placed aside. In addition, we could compare when the placement of the tool directly facilitated confederate beginning-state comfort to when it required participant manipulation to facilitate confederate beginning-state comfort (see Figure 1).

### Data analysis

**Figure 2:**
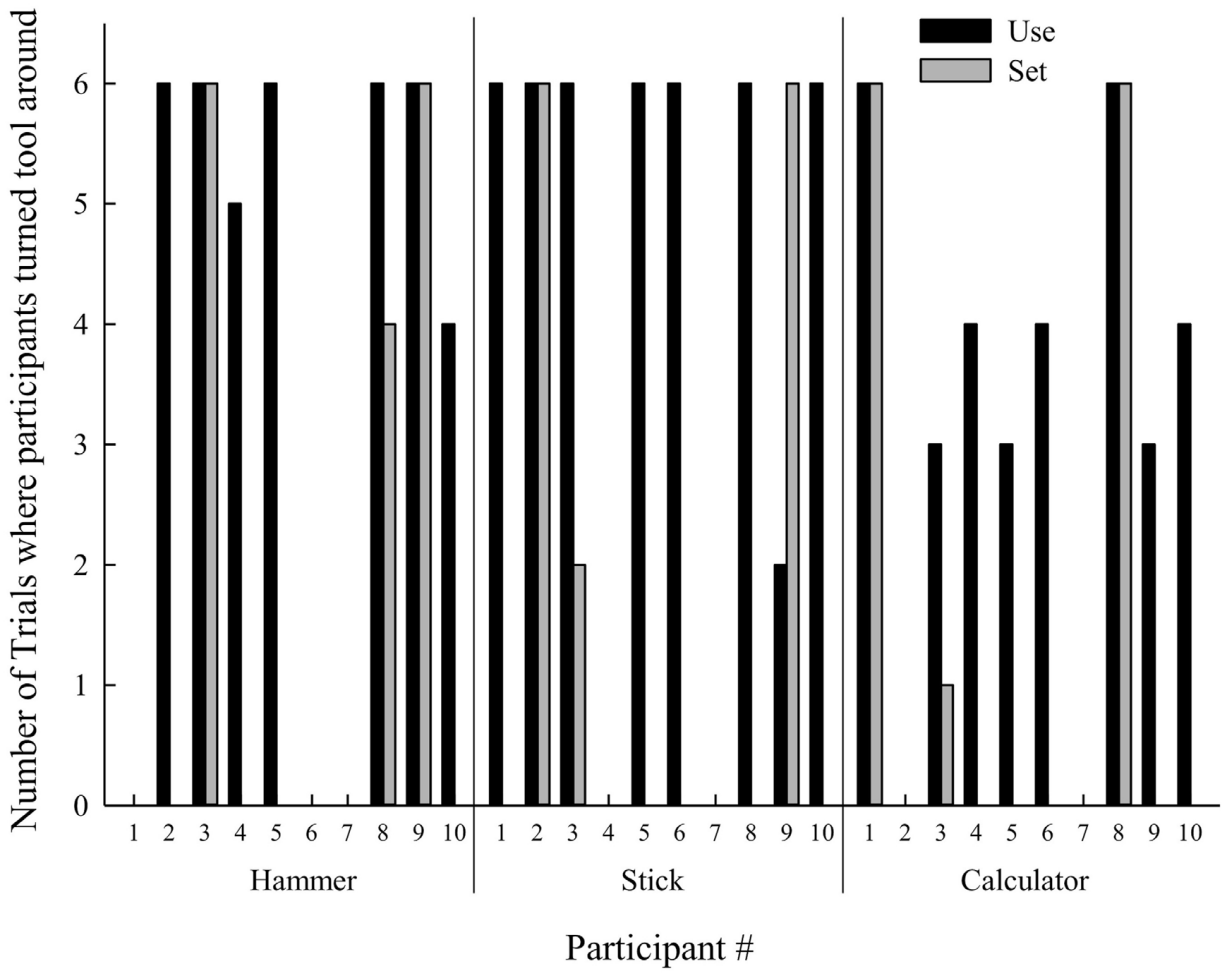
**Number of trials when the tool required manipulation to facilitate the confederate's beginning-state comfort.** For instances where there is no bar, the participant never turned the tool around for the confederate.

The video recordings were reviewed to determine which hand participants used to complete the task and to confirm preference for handedness. The location a participant placed the tool was categorized as contralateral or ipsilateral hemispace relative to the hand they used to pick up the tool. Ipsilateral and contralateral space was used to account for left-handed responses, (i.e., ipsilateral placement would be a contralateral placement for right-handed responses). The final arm orientation was categorized into a comfortable or uncomfortable posture to determine if individuals exhibited end-state comfort (Rosenbaum and Jorgensen, 1992). This was defined by the thumb pointing outwards, or away from the body when using the tool. In addition, beginning-state comfort of the confederate was measured, to determine if the confederate was afforded a comfortable or an uncomfortable initial grasp. It should be noted that the above variables are not continuous and the responses were not normally distributed, therefore parametric statistical tests were not used. Non-parametric tests were not used because the data is not completely binary (which ruled out Cochran's *q*) and the distribution of responses was such that there were too many cells with a count less than 5, which ruled out chi-square. Please see Figure 2 for an illustration of the distribution of responses. Finally, Spearman's correlations were calculated using verbal age/non-verbal ability and the number of times that the participant turned the tools around in order for the confederate to have beginning-state comfort.

## Results

### Self-tasks

#### Hand used

As illustrated in Table 2, the individuals with ASD used their dominant hand for 80% or more of trials for all except one task (Calculator, Set). The TD participants, reported by Gonzalez et al. (2011), used their dominant hand 100% of the time.

**Table 2:** **Percentage (%) of trials participants used dominant hand**.

**Tool**	**Orientation**	**Action**	**Self**	**Other – Hand**	**Other – Place**
Hammer	Uncomfortable	Set	80 (35)	88 (31)	85 (34)
		Hammer	90 (32)	80 (42)	88 (32)
	Comfortable	Set	80 (35)	88 (31)	83 (33)
		Hammer	90 (32)	80 (42)	80 (42)
Calculator	Uncomfortable	Set	95 (16)	100 (0)	100 (0)
		Calculate	82 (39)	100 (0)	100 (0)
	Comfortable	Set	88 (25)	100 (0)	97 (11)
		Calculate	100 (0)	100 (0)	98 (5)
Stick	Uncomfortable	Set	80 (35)	90 (32)	85 (34)
		Hammer	85 (31)	90 (32)	90 (32)
	Comfortable	Set	78 (34)	90 (32)	85 (34)
		Hammer	85 (34)	90 (32)	80 (42)

Standard deviations are reported in brackets.

#### Side placed

As shown in Table 3, when the individuals with ASD placed the tools on one of the two sheets they chose to place the tools almost equally across both sides. TD individuals opted for ipsilateral movements 81% of the time (Gonzalez et al., 2011).

**Table 3:** **Percentage (%) of trials participants placed the tool on the contralateral side**.

**Tool**	**Orientation**	**Self**	**Other – Place**	**Other – Use**
Hammer	Uncomfortable	53 (26)	48 (25)	87 (19)
	Comfortable	57 (26)	57 (29)	92 (14)
Calculator	Uncomfortable	55 (29)	47 (30)	52 (44)
	Comfortable	42 (31)	57 (30)	63 (44)
Stick	Uncomfortable	53 (27)	55 (29)	78 (34)
	Comfortable	53 (13)	55 (28)	87 (25)

Standard deviations are reported in brackets.

#### End-state comfort

Table 4 illustrates the percentage of trials that individuals with ASD demonstrated end-state comfort. For the Self-task, participants demonstrated end-state comfort on 90% or more of trials, except for the calculator–calculate (53%). TD participants demonstrated end-state comfort on 100% of trials for all tools for both the use and place conditions (Gonzalez et al., 2011).

**Table 4:** **Percentage (%) of trials participants demonstrated end-state comfort**.

**Tool**	**Orientation**	**Action**	**Self**	**Other – Hand**	**Other – Place**
Hammer	Uncomfortable	Set	90 (32)	90 (32)	90 (32)
		Hammer	100 (0)	90 (32)	75 (41)
	Comfortable	Set	90 (32)	83 (36)	90 (23)
		Hammer	100 (0)	73 (44)	80 (42)
Calculator	Uncomfortable	Set	98 (5)	100 (0)	90 (16)
		Calculate	53 (48)	100 (0)	98 (5)
	Comfortable	Set	90 (32)	90 (26)	97 (11)
		Calculate	100 (0)	77 (33)	70 (39)
Stick	Uncomfortable	Set	90 (26)	92 (26)	97 (7)
		Hammer	100 (0)	97 (7)	95 (11)
	Comfortable	Set	93 (21)	87 (32)	98 (5)
		Hammer	100 (0)	65 (46)	68 (48)

Standard deviations are reported in brackets.

### Working with other task

#### Hand used

Individuals with ASD used their dominant hand when handing over the tool to the confederate for 80–100% of trials (Table 2). TD participants used their dominant hand for 100% of trials for most conditions (Gonzalez et al., 2011).

#### Side placed

The individuals with ASD chose to place the hammer on their contralateral side on most trials, regardless of the initial orientation (Table 3). TD participants also demonstrated this pattern of performance (Gonzalez et al., 2011).

#### End-state comfort

Individuals with ASD demonstrated end-state comfort ranging from 65 to 100% of trials (Table 4). Overall, end-state comfort was lower when the tool required manipulation because it was initially in a comfortable orientation for the participant. Overall, the participants with ASD also exhibited high between person variability across these conditions.

#### Beginning-state comfort for confederate

**Figure 3:**
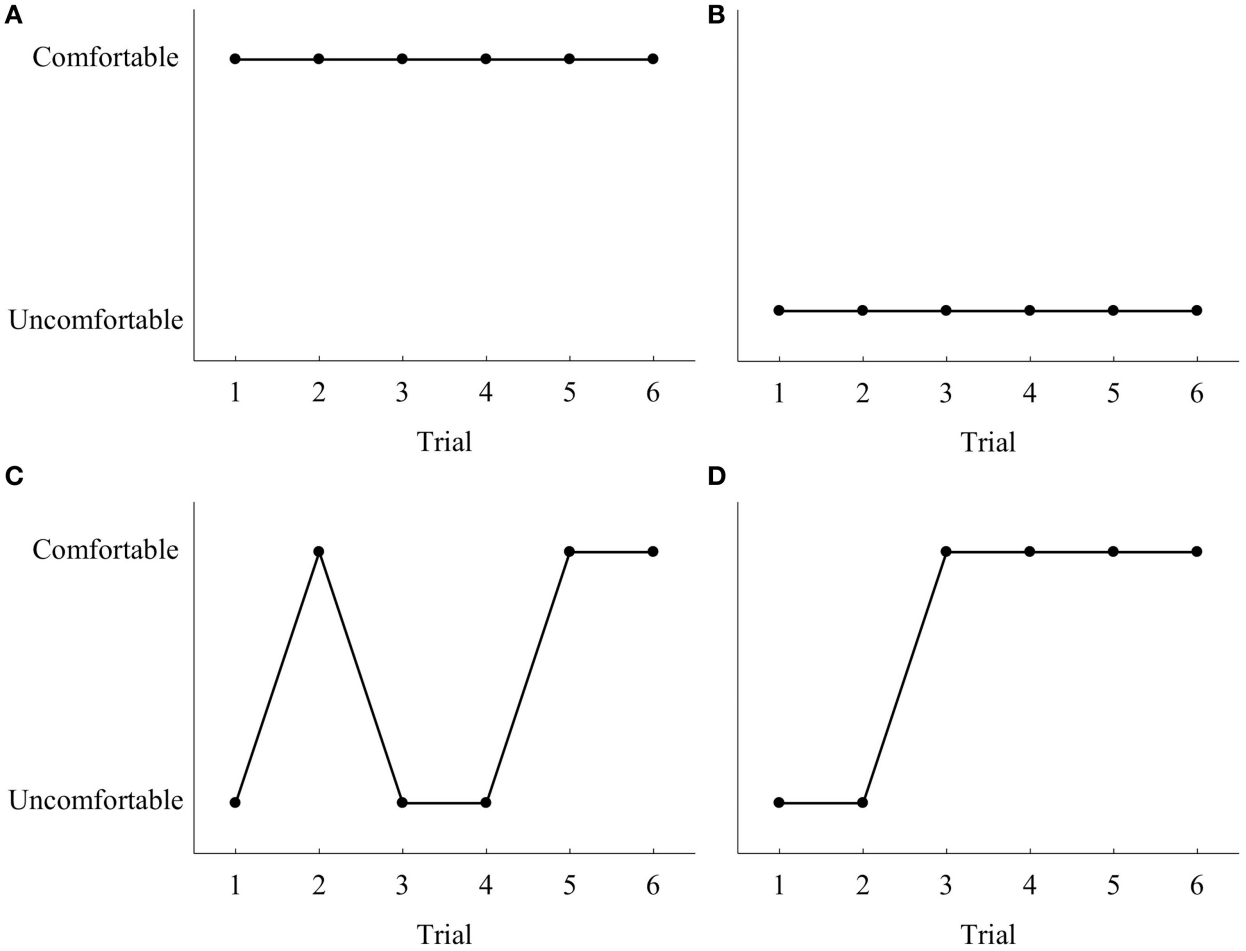
**Sample of the four different types of behavior that were evident in our data when the calculator (chosen due to most variability) was placed in a comfortable position relative to the participant for the tool use condition only. (A)** The participants gave the tool always in manner that facilitated beginning-state comfort for the confederate for both use and set conditions (2 participants). **(B)** The participants always passed the tool in a manner that did not facilitate beginning-state comfort (2 participants). **(C)** The participants changed their strategy of handing the tool over to the confederate inconsistently (3 participants). **(D)** The participants changed their strategy to handing the tool over to the confederate in a manner that facilitated beginning-state comfort (3 participants).

When asked to hand the tools to the confederate so that he could *use* the tool, participants with ASD oriented the tool (when placed in a comfortable position in relation to the participant) in a manner that allowed the confederates to adopt a comfortable beginning-state posture in most instances (Table 5). Furthermore, when the confederate did not use the tool, the percentage of trials that ASD participants facilitated the confederate's beginning-state comfort decreased (Table 5, Figure 2). However, participants exhibited considerable between person variability. Figure 3 illustrates the variability in the patterns observed by plotting participants' individual performance across trials for the calculator (the calculator had the most within participant variability). Further inspection of Figure 3 indicates that some individuals with ASD handed the tools in a manner that benefited the confederate, although it inconvenienced their own posture (i.e., either beginning-state or end-state discomfort). However, the trial-by-trial graphs for these conditions show that the individuals with ASD did not always adopt the same strategy for passing tools. Furthermore, no strategies describe the performance of all the participants. It is of interest that there was more variability in strategy when the confederate was going to use the tool, as when the confederate was not going to use the tool, only two strategies were observed (100% comfortable or 0% comfortable beginning-state comfort for confederate, not plotted). Only one participant exhibited a change of strategy when the confederate was to place the tool down.

**Table 5:** **Percentage (%) of trials that confederate received tool in comfortable manner during working with other tasks**.

**Tool**	**Orientation**	**Action**	**Hand**	**Place**
Hammer	Uncomfortable	Set	88 (31)	78 (42)
		Hammer	97 (7)	87 (32)
	Comfortable	Set	27 (44)	12 (31)
		Hammer	65 (46)	48 (51)
Calculator	Uncomfortable	Set	100 (0)	85 (32)
		Calculate	100 (0)	97 (7)
	Comfortable	Set	22 (42)	10 (32)
		Calculate	55 (34)	53 (48)
Stick	Uncomfortable	Set	80 (42)	82 (38)
		Hammer	80 (38)	67 (47)
	Comfortable	Set	23 (42)	2 (5)
		Hammer	73 (44)	68 (44)

Standard deviations are reported in brackets.

#### Correlations for beginning-state comfort of confederates

No significant correlations were found when Spearman correlations between verbal age scores, IQ equivalent scores, and performance on handing the tools in a comfortable beginning-state for the confederate were performed (*p* > 0.05). Specifically the correlation *S* for verbal age and the hammer was 0.30, for verbal age and stick was −0.29, and for verbal age and calculator was −0.31. The correlations between IQ equivalent scores were generally higher (0.30 for hammer, 0.40 for stick, and 0.56 for calculator).

## Discussion

The main purpose of the present study was to assess whether individuals with ASD consider the motoric perspectives of another individual and plan their own movements to facilitate the performance of another person. We adopted the same paradigm used in the Gonzalez et al. (2011) paper, in which we asked participants to pass tools to a confederate so the confederate could accomplish a motor task (e.g., hammer a peg). Participants planned their movements to account for their own comfort at the end of the movement for the majority of trials (65–100%), demonstrating they can plan their movements in advance when the movement requires interpersonal interaction. With respect to consideration of the other actor's comfort, *overall* the group of participants with ASD considered the perspective of the other person and planned their actions to facilitate the beginning-state comfort of the confederate. That said, individuals with ASD demonstrated considerably more variations both within and between individuals as compared to previous literature in the TD population (Rosenbaum and Jorgensen, 1992; Gonzalez et al., 2011). We believe that using this joint-action paradigm may be a valid method to test the fundamental behavior underlying ToM because a verbal response is not required to be successful at the joint-action task. The clear between person variability may also provide novel methods for assessing subgroups of individuals with ASD.

Gonzalez et al. (2011) demonstrated that TD participants consider the intended action of a confederate and plan their actions accordingly, which we suggest is indicative of the ability to use ToM in this paradigm. That is, when the confederate was going to use the tool, the TD participants handed the tools in a manner that facilitated beginning-state comfort for the confederate on 100% of the trials (Gonzalez et al., 2011). In contrast, when a participant was asked to hand the tool to the confederate, who was *not* going to use the tool, the percentage of times the participant adopted beginning or end-state discomfort decreased (63% for hammer, 10% for stick, and 25% for calculator). This change in behavior demonstrates that the TD participants considered what the confederate was going to do with the tool and adjusted their behavior accordingly.

Participants with ASD displayed a range of behaviors which resulted in greater between person variability than their TD peers. As illustrated in Table 4, individuals with ASD demonstrated a tendency toward end-state comfort (cf. calculator—calculate), however, not all participants behaved in the same manner. By comparison, TD participants demonstrated end-state comfort on 100% of the trials for all tools for both the use and place conditions (Gonzalez et al., 2011). Much larger within person variability was also evident when working with the calculator, which we believe reflects our prediction that the intended action of the calculator was more subtle than the hammer or hammering with the stick. Consistent with Gonzalez et al. (2011), a subgroup of the participants with ASD perceived the end goal of the confederate and planned their movements to maximize his beginning-state comfort (i.e., they turned the tool to allow the confederate to use the tool without further manipulation). Therefore, a subgroup of individuals with ASD successfully coordinated their actions with those of another so the overall goal could be achieved in a more efficient manner.

We also predicted that participants' performance would improve when the physical characteristics of the tool directly prompted its correct use (i.e., hammer > stick). In contrast, we found that participants manipulated objects in order to facilitate the confederate's end-state comfort more often when the object exhibited physical characteristics that did not directly prompt its correct use (stick > hammer). In retrospect, the task of hammering with the stick appeared to facilitate efficient movement planning when compared to the hammer perhaps because the participant did not have to override his/her urge to grasp the hammer by the handle rather than head, which would have been necessary in order to turn it around so that it was graspable for the confederate. In addition, when passing the stick the added instruction regarding which end would be used for hammering could have facilitated movement planning. In contrast, the calculator, whose physical characteristics arguably had the least direct relationship with the action, was only manipulated by the participant 55% of the time when doing so was necessary for the confederate to achieve beginning-state comfort. This finding indicates that motor planning was improved for joint-actions when the more direct physical characteristics of the object better matched the task goal (stick and hammer > calculator). The latter result is consistent with prior movement planning literature demonstrating that individuals with ASD use direct visual information to plan their movements. In line with the present results, their performance differs when the task requires more complex planning behavior (Glazebrook et al., 2008). To the best of our knowledge, this is some of the first empirical evidence to demonstrate that individuals with ASD can coordinate their actions with another person when they share a common goal.

On a more individual level, we found that joint-action behaviors were less straightforward for individuals with ASD than for TD individuals. Specifically, 2 participants always turned the tool around to ensure comfortable beginning-state comfort for the confederate, while three other participants changed their strategy after one or two trials to facilitate the beginning-state comfort of the confederate. Three different participants appeared to change their strategy randomly, and two individuals never passed the tool in a comfortable manner for the confederate. In other words, individual participants adopted a variety of strategies and therefore no “typical” strategy was evident for individuals with ASD.

Of note is that no individual changed his or her strategy when the tool was not going to be used by the confederate (i.e., place condition). Two participants always oriented the tool in a comfortable manner for the confederate, regardless of whether the confederate was going to use the tool or not. We propose that these two participants had learned a “rule” that they applied regardless of context. For the other eight participants, it was not as straightforward to decipher why they never oriented the tool for the confederate to have beginning-state comfort in the place condition. Some participants may simply not consider that re-orienting the tool will benefit the confederate. This is the most probable explanation for those participants who never re-oriented the tool to a comfortable position for the confederate. Alternatively, this sub-group of participants could have been fully aware that the confederate would not use the tool and therefore the orientation did not matter.

We also found that, similar to TD participants, individuals with ASD preferred to use their dominant hand for the majority of trials (80–100%). However, unlike TD participants who placed the tool in ipsilateral space most of the time (80% or more), individuals with ASD placed the tool in ipsilateral and contralateral space equally often (42–57%), except when the confederate was going to use the tool. Because reaching across the body requires a longer reach, economy of movement may not be a priority for individuals with ASD. This pattern of behavior is consistent with the idea that individuals with ASD plan basic movements successfully but do not incorporate advanced variables, such as location within the environment, into their movement plan. The variability individuals with ASD experience in movement planning and control (Glazebrook et al., 2006, 2009) would make action planning more difficult. Thus, reducing the number of variables to consider (i.e., location in the environment) may help to simplify the motor task.

Our findings are consistent with van Swieten et al. (2010) who reported that children with ASD performed similar to their age matched TD peers. Although some evidence of motor planning was evident, the large variability in the ASD participants is in line with other research (Hughes, 1996) that shows individuals with ASD demonstrate lower end-state comfort, even when compared to younger TD children. As mentioned before, this could be a function of the wide range of abilities found in the ASD population. Careful consideration should be taken when looking at group performance. Instead, we believe that considering the different pattern of behaviors may provide more insight than a group norm. Indeed, links between motor adaptability and severity of more traditional symptoms of ASD have been reported (e.g., Haswell et al., 2009).

Our purpose for this initial study was to test the relevance and feasibility of this novel interpersonal coordination task. We acknowledge that our sample size is relatively small and that there is great variability across the ASD population for most tasks, including the ability to solve ToM problems. Indeed, half of our participants demonstrated an ability to successfully act or change their strategy to aid the confederate in acquiring the goal. Thus, this new interpersonal coordination task may tap into a fundamental skill that relies on non-verbal communication and can be taught using direct motor interactions. Future work will continue to develop the links between motor performance and deficits in social and communication behaviors by directly comparing performance of this task with ToM and joint attention abilities. Extending the results of the present study will help to establish how early motor skills contribute to the development of behaviors such as interpersonal coordination and joint attention.

We believe that the tasks reported here provide a novel method to assess an individual's ability to plan his/her movements in two specific contexts: (1) one that requires consideration of their own performance only; (2) one that requires consideration of the performance of a partner. The latter may be used to test ToM behaviors in a novel way, that is, without requiring a verbal response. If it is true that internal action models are a necessary step for understanding intentions (Mostofsky and Ewen, 2011), then perhaps individuals who exhibit the ability to solve joint-action problems may also have better success learning more complex social interactions. Therefore, a joint-action task could also be used as a novel method for training individuals with ASD to plan their own actions in the context of another, thereby providing a link between fundamental and more complex interpersonal interactions.

### Conflict of interest statement

The authors declare that the research was conducted in the absence of any commercial or financial relationships that could be construed as a potential conflict of interest.
